# Sense of agency and addiction

**DOI:** 10.3389/fpsyg.2026.1725049

**Published:** 2026-03-31

**Authors:** F. Gregory Ashby, Graham Z. Ashby

**Affiliations:** 1 Department of Psychological & Brain Sciences, University of California, Santa Barbara, CA, United States; 2 Stillwater Behavioral Health, Montecito, CA, United States

**Keywords:** addiction, agency, compulsion, craving, dopamine, relapse

## Abstract

Agency is the sense that one has control over one's own actions and the consequences of those actions. A recent theory proposes that increases in agency disinhibit the dopamine system and thereby increase the number of tonically active dopamine neurons in the ventral tegmental area. The theory, called ADDS (Agency Disinhibits the Dopamine System), proposes a specific neural network that mediates these effects. ADDS successfully accounts for a variety of relevant neuroscience and behavioral results. Novel predictions are derived from ADDS about how the sense of agency impacts all aspects of drug addiction, including (1) the acquisition and maintenance of addictive behaviors; (2) cravings, compulsions, and relapse; and (3) treatment and recovery. ADDS predicts that increases in agency will increase the user's motivation to find and take drugs, and accelerate social drug taking. The theory also predicts that increases in agency will strengthen the antireward response that follows a drug high, and therefore exacerbate cravings and relapse. As a result, the theory predicts that treatment of substance-use disorders may be facilitated by appropriate changes to the client's sense of agency. The most therapeutic approach might be to elevate the client's agency at some times and weaken it at others.

## 1 Introduction

Agency is the sense that one has control over one's own actions and the consequences that result from those actions (Moore, 2016). An enormous literature suggests that agency plays a critical role in a wide variety of human behaviors (e.g., Bandura, 2006; Mele, 2003; Russell, 2013) and in the efficacy of the psychotherapeutic process (e.g., Bandura, 1977).

Recently, progress has been made in understanding the neural consequences that occur when agency increases or decreases. Specifically, Ashby et al. (2024) proposed that increases in agency disinhibit the dopamine (DA) system and thereby increase the number of tonically active DA neurons in the ventral tegmental area (VTA). The theory, called ADDS (Agency Disinhibits the Dopamine System), proposes a specific neural network that mediates these effects and it accurately predicts a variety of relevant neuroscience and behavioral results. This article extends ADDS to addictive behaviors by deriving many novel predictions about how the sense of agency impacts all stages of addiction, with a focus on the role that agency plays in (1) the acquisition and maintenance of addictive behaviors; (2) cravings, compulsions, and relapse; and (3) treatment and recovery.

## 2 The ADDS theory of agency

**Figure 1:**
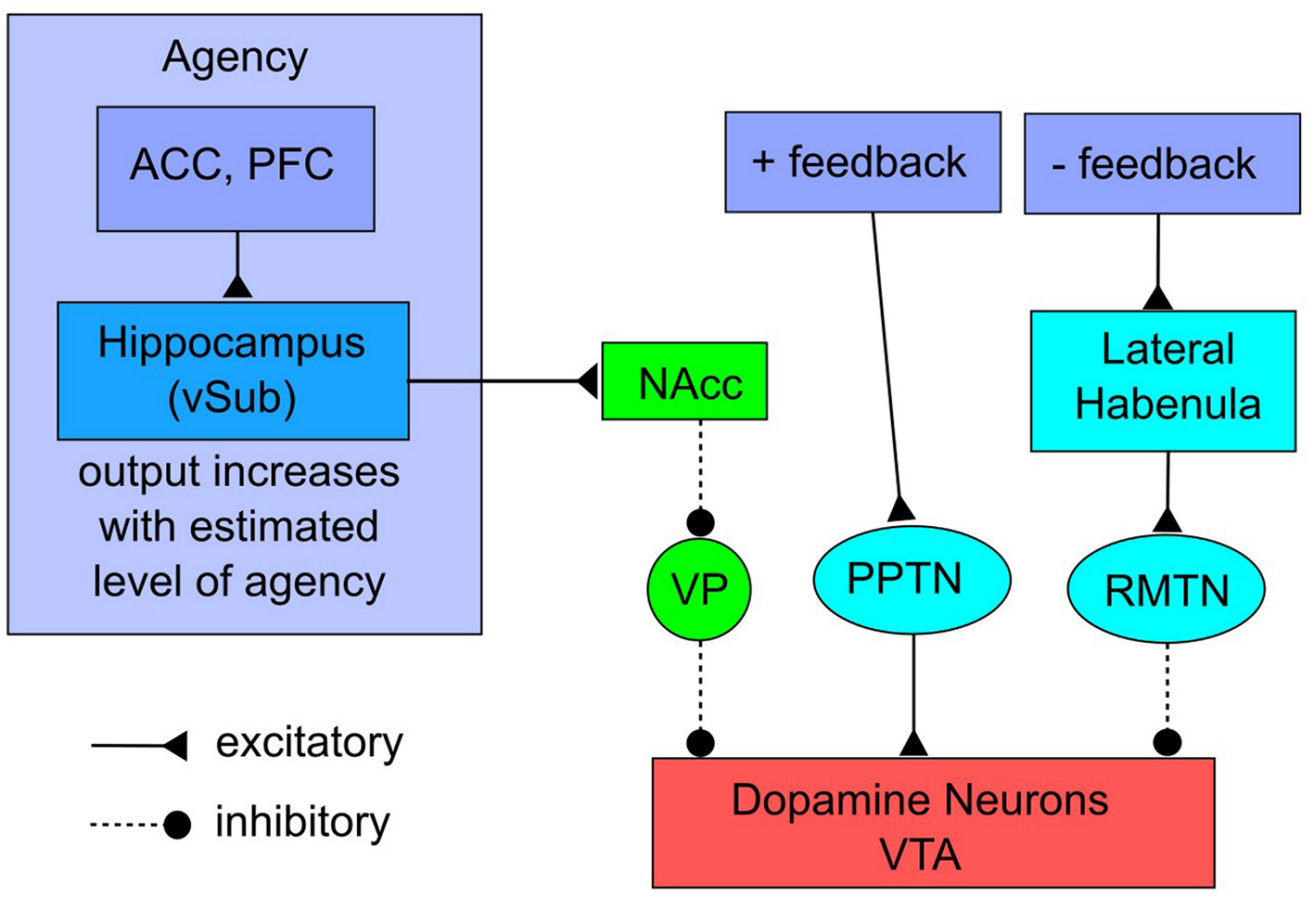
The ADDS theory of how agency modulates the firing of DA neurons. ACC: anterior cingulate cortex; PFC: prefrontal cortex; vSub: ventral subiculum; NAcc: nucleus accumbens; VP: ventral pallidum; PPTN: pedunculopontine tegmental nucleus; RMTN: rostromedial tegmental nucleus; VTA: ventral tegmental area. Adapted from (Inglis et al. 2021).

The neural circuits that underlie the ADDS model are described in Figure 1. For justification of this model, more detail, and many empirical tests of the model's validity, see Ashby et al. (2024). The right half of the network instantiates a standard model of how positive and negative feedback affect the firing of DA neurons in the VTA. Considerable evidence shows that an unexpected reward causes reward-sensitive neurons in regions such as prefrontal and orbitofrontal cortices to provide excitatory inputs to the pedunculopontine tegmental nucleus, which stimulates the DA neurons in the VTA and causes the tonically active VTA DA neurons to fire phasic bursts (Hong and Hikosaka, 2014; Kobayashi and Okada, 2007; Okada and Kobayashi, 2013). This phasic firing raises DA levels in all VTA target regions. In contrast, if an expected reward fails to appear, then other reward-sensitive neurons stimulate the lateral habenula, which stimulates the rostromedial tegmental nucleus, which inhibits the tonically active VTA DA neurons, thereby lowering DA levels in all VTA targets (Tian and Uchida, 2015; Hong et al., 2011; Matsumoto and Hikosaka, 2007, 2009).

The left half of Figure 1, which is more novel, describes a network via which changes in the sense of agency disinhibit the VTA DA neurons. The evidence is good that people continuously monitor their own agency, and are quick to note, for example, when environmental conditions change in a way that increases or decreases their agency (Metcalfe and Greene, 2007). The model assumes that estimates of agency are continuously updated by a network that includes regions in the anterior cingulate, prefrontal cortex, and hippocampus, and that the outputs of this network gate the amount of DA release via projections through the hippocampal ventral subiculum (vSub; via projections proposed by Grace et al., 2007).

The projections from vSub to the nucleus accumbens are excitatory and the projections from the accumbens to the ventral pallidum and from the ventral pallidum to the DA neurons of the VTA are inhibitory. Even so, a key feature of this neuroanatomy is that the tonic firing rate of ventral pallidal neurons is much higher than the tonic firing rate of nucleus accumbens neurons. As a result, many DA neurons in VTA are silent due to tonic inhibition by the ventral pallidum. Estimates suggest that because of this inhibition, only about half of VTA DA neurons are spontaneously active under control conditions, and these tonically firing neurons are the only ones available to respond to rewards (Grace et al., 2007; Lodge and Grace, 2006). ADDS predicts that when agency is high, vSub excites the nucleus accumbens, which inhibits the ventral pallidum. This releases VTA DA neurons from tonic inhibition, which increases the number of tonically firing VTA DA neurons, thereby raising tonic DA levels in all VTA target brain regions and enlarging the pool of DA neurons that can respond to rewards. In contrast, if agency suddenly drops, ADDS predicts that the vSub excitation of the nucleus accumbens will decrease, which reduces inhibition on the ventral pallidum, and that the resulting increase in pallidal activity will increase inhibition of the VTA DA neurons, thereby reducing the number that are tonically active.

ADDS therefore makes two fundamental and novel neuroscience predictions. First, increases in agency should increase tonic DA levels in all VTA target regions (e.g., frontal cortex). Second, increases in agency should increase the number of DA neurons available to respond to feedback and thereby amplify the DA response to positive and negative feedback. Furthermore, these predictions are causal in the sense that ADDS predicts that any increase in agency, no matter what the cause, will lead to these predicted DA effects.

Inglis et al. (2021) built a computational model of the Figure 1 network that was constructed from mathematical models of spiking neurons, and they showed that this model accurately predicts a variety of relevant neuroscience results.^1^ For example, it provides a good quantitative account of the differential effects on average firing rate and on the number of tonically active DA neurons in the VTA that result from stimulating the ventral subiculum, the pedunculopontine tegmental nucleus, or both (i.e., data reported by Lodge and Grace, 2006). These and other results reported by Inglis et al. (2021) support the validity of the specific neural network illustrated in Figure 1, which was used to derive all of the behavioral predictions of ADDS.

ADDS also makes many novel and testable behavioral predictions that have strong preliminary empirical support (for details, see Ashby et al., 2024). This includes predictions that increases in agency will (1) increase motivation, (2) improve all forms of executive function, (3) facilitate procedural learning, but only in the presence of immediate trial-by-trial feedback, (4) have little or no effect on learning-related effects due to perceptual priming or on the acquisition or expression of standard-eyeblink conditioning, (5) facilitate the development of automatic behaviors, but have little or no effect on the production of behaviors that are already automatized, (6) amplify the cognitive benefits of positive affect, and (7) reduce pain.

There is considerable overlap between the Figure 1 network and the networks that support addiction, which is partly why ADDS makes many strong predictions about how agency affects all stages of addiction. First, a popular proposal is that addictive drugs interact in some way with the DA system (e.g., Lüscher and Janak, 2021). For example, virtually all addictive drugs have been shown to increase DA levels in the nucleus accumbens of rats (Di Chiara and Imperato, 1988). Second, electrical stimulation of vSub reinstates cocaine and amphetamine seeking in rats after it has been extinguished, and this reinstatement is blocked if the nucleus accumbens is prevented from responding in its normal way or if the VTA is inhibited (Taepavarapruk and Phillips, 2003; Taepavarapruk et al., 2015; Vorel et al., 2001). Third, this same vSub → accumbens pathway has been implicated in the reinstatement of both heroin and alcohol seeking in rats following re-exposure to cues that were present during initial drug addiction (Bossert and Stern, 2014; Marchant et al., 2016).

## 3 Agency and the acquisition of addictive behaviors

Koob and Le Moal (2008a) proposed that drug taking elicits two opposing processes. Initially, the reward system is activated, which elevates the hedonic state and mediates the rewarding properties of the drug (i.e., the “high”). Shortly after this hedonic high begins to decay back to baseline, an antireward system is activated that opposes the positive hedonic state induced by the reward system. The antireward system induces a negative hedonic state that includes “chronic irritability, emotional pain, malaise, dysphoria, alexithymia, and loss of motivation for natural rewards” (Koob and Le Moal, 2008a, p. 38). There is considerable independent evidence supporting this opponent-process model, both in rodents (Broderick et al., 1984; Gardner and Lowinson, 1993; Karin et al., 2021; Nazzaro et al., 1981) and humans (Gardner and Lowinson, 1993; Hand et al., 2024; Solomon and Corbit, 1974).

Evidence suggests that with extended use, the reward response weakens and the antireward response strengthens (Koob and Le Moal, 2008a). For example, down-regulation of DA receptors reduces the overall efficacy of any fixed drug dose. As a result, larger doses are required to achieve the same high. In contrast, the strengthening of the antireward response leads to the chronic negative-hedonic states described above. In the majority of social drug users, these negative-hedonic states are sufficient to abolish drug seeking. Around 15% of social-drug users though, persist in drug taking, despite these negative consequences (Anthony et al., 1994). Curiously, the percentage is similar in rats. For example, almost all rats will learn to self-administer cocaine, but only around 17% will persist when the cocaine delivery is accompanied by a punishment (e.g., an electric shock; Deroche-Gamonet et al., 2004). Once drug taking persists despite negative consequences, it is defined operationally as a compulsion (Lüscher and Janak, 2021). At this point, drug taking not only elicits the rewarding properties of the drug, but it also alleviates the chronic negative-hedonic state.

The reward system includes regions of prefrontal cortex, hippocampus, nucleus accumbens, and the basolateral amygdala (e.g., Lüscher and Janak, 2021), whereas the antireward system includes medial prefrontal cortex, the central nucleus of the amygdala, and the bed nucleus of the stria terminalis (Koob and Le Moal, 2008b). All of these brain regions receive a prominent DA projection from the VTA, and ADDS predicts that increases in agency should increase the number of tonically active DA neurons in the VTA. As a result, ADDS predicts that increases in the sense of agency will affect both the reward and antireward response to drugs. These effects will be of two types. First, an increase in agency will increase the user's motivation to find and take drugs because motivation is thought to increase with tonic DA levels (Niv et al., 2007; Salamone et al., 1994). Second, DA facilitates synaptic plasticity (e.g., Jay, 2003), and therefore increases in agency should accelerate synaptic plasticity in these regions and therefore accelerate social drug taking as well as the strengthening of the antireward response.

## 4 The role of agency in craving, compulsion, and relapse

A craving is an intense desire to consume a drug, whereas a compulsion is a repetitive urge in which an individual feels compelled to consume a drug, even when it is known that there will be harmful consequences (American Psychiatric Association, 2022). A craving is typically associated with a desire for the drug reward, whereas a compulsion is often associated with a goal of reducing anxiety or distress. Craving and compulsion are difficult to study in nonhuman animals since they are internal states rather than behaviors. Nevertheless, some widely accepted operational definitions have led to many nonhuman animal studies of craving and compulsion. Specifically, a widely used behavioral criterion is that a craving exists if, following withdrawal, one or more cues associated with drug taking induce drug-seeking behaviors (e.g., Shaham et al., 2003). In contrast, a compulsion is said to exist if an animal continues to self-administer a drug in the presence of an aversive or punishing stimulus, such as foot shock (Deroche-Gamonet et al., 2004; Lüscher and Janak, 2021). It is important to acknowledge, however, that these operational criteria are unlikely to capture the human conditions of craving and compulsion perfectly (as described, e.g., by the DSM-5-TR). As a result, inferences about human craving and compulsion drawn from animal models must be interpreted with caution.

**Figure 2:**
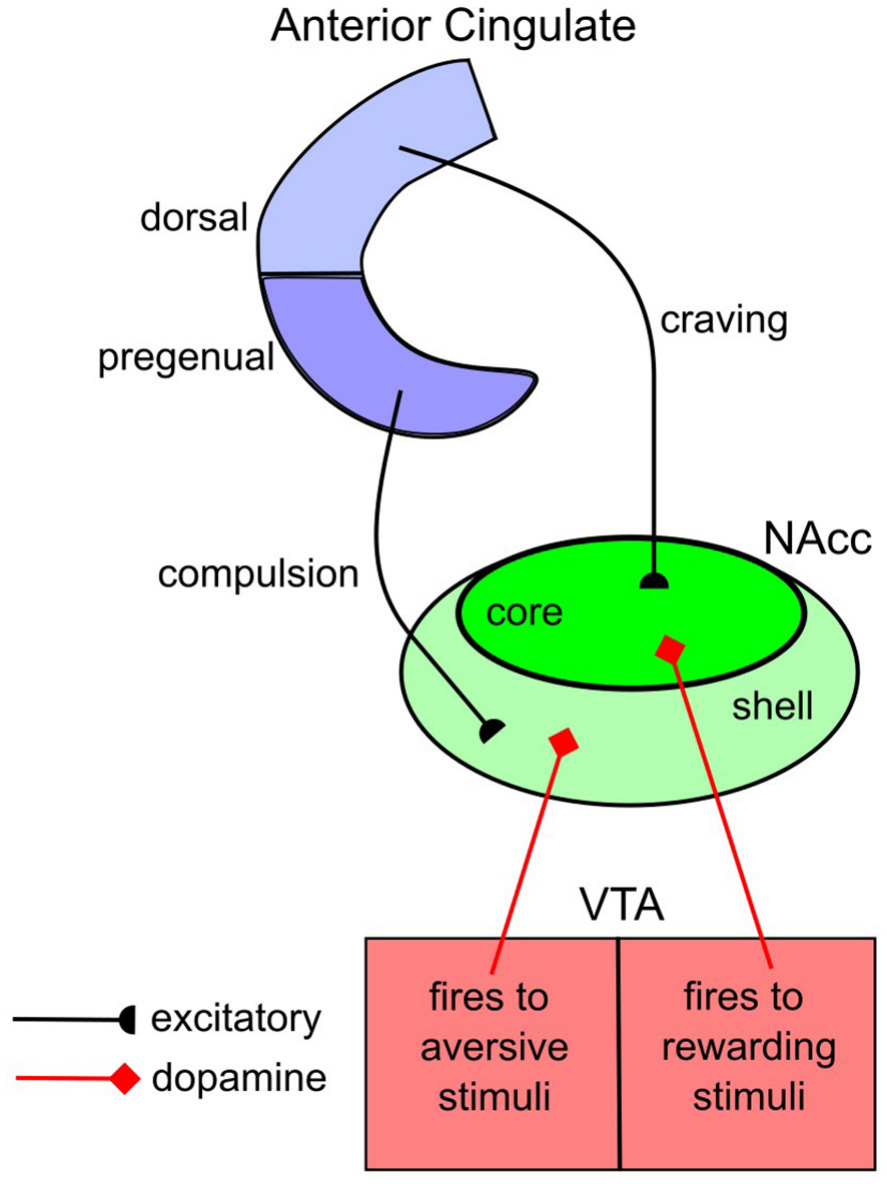
A simplified model of how projections from medial PFC to the nucleus accumbens contribute to craving and compulsion. NAcc: nucleus accumbens; VTA: ventral tegmental area.

Many widely distributed brain regions contribute to drug craving and compulsion, including for example, orbitofrontal cortex, medial prefrontal cortex (mPFC), anterior cingulate, insula, and striatum (e.g., Robbins et al., 2024). A simplified model that focuses on key projections is illustrated in Figure 2. The literature supporting this model comes from both rat and human studies, and unfortunately, the neuroanatomical naming of some key brain regions is different in the two species. In the rat, the focus has been on projections from the mPFC to the nucleus accumbens. The region of the mPFC most closely tied to craving is the prelimbic cortex, which is homologous to the dorsal anterior cingulate (dACC) in humans, and the cortical region most closely tied to compulsion is the infralimbic cortex, which is homologous to the pregenual anterior cingulate (pgACC) in humans. We discuss each of these projections in turn.

Many studies with rats support the Figure 2 model of drug craving (e.g., McGlinchey et al., 2016; Moorman and Aston-Jones, 2023; Zhao et al., 2025). For example, cocaine self-administration strengthens synapses between prelimbic cortex (dACC) and the NAcc core and these synapses seem to be critical for craving because artificially weakening the same synapses 45 days after withdrawal (via optogenetics) reduces cue-induced cocaine seeking (Ma et al., 2014). In contrast, artificially weakening synapses between infralimbic cortex (pgACC) and the NAcc has the opposite effect—that is, it increases drug-seeking behaviors (Ma et al., 2014). Furthermore, there is also evidence that the prelimbic → NAcc (dACC → NAcc) core projections are sensitive to interoceptive, rather than exteroceptive drug cues, as predicted by the craving hypothesis (Randall et al., 2020).

Based in large part on studies of this type, the human dACC has been a key target in human treatments that attempt to reduce drug cravings (e.g., Zhao et al., 2021). For example, repetitive transcranial magnetic stimulation directed at this region reduces alcohol cravings for up to 6 weeks (De Ridder et al., 2011, 2016). Similarly, nicotine cravings are reduced when neurofeedback (i.e., real-time fMRI) is used to reduce activity in the dACC (Li et al., 2013).

ADDS predicts that an increase in agency will increase tonic DA levels in all target areas of VTA DA neurons, which includes all regions of mPFC and the NAcc. DA increases the signal-to-noise ratio at glutamatergic synapses (Ashby and Casale, 2003).^2^ As a result, ADDS predicts that an increase in agency will increase the response in the NAcc core to any given level of craving-associated dACC activation. Therefore, ADDS predicts that increases in agency will exacerbate cravings.

The antireward response that can accompany a compulsion depends critically on projections to the NAcc core and shell from the infralimbic cortex (pgACC). Of these, the projections to the NAcc shell are likely more important since this is also the target of VTA DA neurons that respond to aversive stimuli (Badrinarayan et al., 2012; de Jong et al., 2019). For example, artificially weakening synapses between infralimbic cortex (pgACC) and the NAcc shell increases drug-seeking behaviors in rats (Ma et al., 2014; Nett et al., 2023), theoretically, because it reduces one important barrier to drug seeking—namely, the negative-hedonic state induced by the antireward system.^3^

As one might predict from these rat studies, the human pgACC is arguably the key region associated with mood disorders and especially with major depression (Drevets et al., 2008; Hamani et al., 2011). Activity in this area is elevated during periods of depression and effective antidepressant treatments tend to reduce activity in this region (e.g., Drevets et al., 2008). Furthermore, the pgACC is the region most often targeted via deep-brain stimulation (DBS) in treatment-resistant major depression (Sobstyl et al., 2022). DBS interferes with the ability of the implanted brain region to respond in its usual way, and so functionally, the effects of DBS are similar to a brain lesion (e.g., Chiken and Nambu, 2016). Therefore, the evidence strongly suggests that compulsions in drug addiction are associated with increased activity in the pgACC.

The accumbens response to infralimbic (pgACC) input is balanced by DA D1 and D2 receptor activation, with D1 activation increasing accumbens activation and D2 activation having the opposite effect. Drug addiction changes this picture in two ways. First, drug taking causes abnormal strengthening of the infralimbic → NAcc (pgACC → NAcc) synapses, and second, drug taking causes a disproportionate downregulation of D2 receptors, compared to D1 receptors (Volkow et al., 2007). This selective downregulation occurs because the rewarding properties of drugs, at least within the NAcc, are mediated primarily by D2 receptor activation. The consequences of these two changes will be an abnormally large increase in NAcc activation to the same level of infralimbic (pgACC) activation. Because increased activation in the NAcc shell signals an aversive event, this model accounts for the increased antireward response that develops with addiction.

ADDS predicts that an increase in agency will exacerbate this antireward response. According to ADDS, an increase in agency will elevate tonic levels of DA in the NAcc. But this extra DA will preferentially activate D1 receptors because of the downregulation of D2 receptors, thereby increasing accumbens activation and thus exaggerating the negative hedonic state activated by the antireward system. As a result, ADDS predicts that increases in agency will increase compulsive behaviors.

A glance back at Figure 1 shows that in the ADDS model, the key input region to the NAcc comes from vSub, which is a major output structure in the ventral hippocampus. The ventral hippocampus receives input from dorsal regions of hippocampus, which contain place cells that encode the current location, as well as locations that were visited previously (e.g., Moser et al., 2015). The ventral hippocampus also receives input from limbic regions, including the amygdala (French et al., 2003) and hypothalamus (O'Mara, 2006). One prominent hypothesis is that ventral hippocampus integrates these spatial and limbic inputs and as a result, essentially encodes the emotional significance of current and previous locations (Grace, 2010; Malagon-Vina et al., 2023). This observation is especially relevant to place conditioning, in which an association develops between the environment in which a drug is taken, and the drug itself (for a review, see, e.g., McKendrick and Graziane, 2020). Returning to the drug-taking environment during withdrawal often elicits drug craving and relapse in humans (O'Brien et al., 1992).

There is considerable evidence supporting the hypothesis that this type of drug-related place conditioning is mediated by projections from vSub → NAcc. For example, in a number of studies, rats either self-administered or were given heroin, alcohol, amphetamine, or cocaine in one conditioning environment and then experienced a number of days of withdrawal in a different environment. Using a variety of different techniques, these studies demonstrated that relapse due to place conditioning is associated with selective activation of the vSub → NAcc projection (heroin: Bossert et al., 2016; alcohol: Marchant et al., 2016; amphetamine: Taepavarapruk et al., 2015; cocaine: Caban Rivera et al., 2025).^4^

These studies established a key role for the vSub → NAcc projection in context-induced relapse, but they did not examine the subsequent effects on DA neuron activity. This was the focus of a study in which rats were administered amphetamine daily for 5 days, then given a 5-day withdrawal period (Lodge and Grace, 2008). On the next day, the rats were placed back in the apparatus where they received the amphetamine. During this period, the authors recorded from DA neurons in the VTA. The results showed that a return to the drug-receiving environment did not increase phasic firing of VTA DA neurons, but it did cause the average number of tonically active VTA DA neurons to increase. Furthermore, subsequent tests showed that this increase was caused by increased vSub output. In contrast, when drug naïve animals were administered an acute dose of amphetamine, the number of tonically active DA neurons decreased, relative to control.

Note that these results are identical to the predictions that ADDS makes about the neural consequences of increasing agency. Specifically, the neural effects of returning a drug-addicted animal to the drug-taking environment during withdrawal are identical to the neural effects that ADDS predicts should follow a sudden increase in agency. In both cases, the effects are initially mediated by vSub → NAcc projections and in both cases, the result is an increase in the number of tonically active DA neurons in the VTA. In other words, ADDS predicts that moving from an innocuous location to one previously associated with unexpected rewards will have the same effects on VTA DA neuron firing as a sudden increase in the sense of agency.

What are the implications of these predictions for the treatment of drug addiction? First, we know that a return to the drug-taking environment frequently induces craving and relapse. Second, the evidence is also good that these effects are initiated by increased activation of the NAcc by vSub. For example, electrical stimulation of vSub, but not cortex, caused reinstatement of amphetamine self-administration following withdrawal in rats (Taepavarapruk et al., 2015). Third, the evidence is also good that activation of the vSub → NAcc pathway increases the number of tonically active DA neurons (Lodge and Grace, 2006, 2008). Fourth, there is overwhelming evidence that an increase in the number of tonically active DA neurons has profound effects on motivation, executive function, learning, and memory (e.g., Ashby et al., 2024). An obvious hypothesis that follows from these observations is that any factor that increases activation on the vSub → NAcc pathway after re-entering the drug-taking environment during a period of withdrawal or recovery will exacerbate drug craving and relapse. ADDS predicts that activity on this pathway should increase with the client's sense of agency. So the higher the sense of agency when the drug-taking environment is revisited, the higher the activation on the vSub → NAcc pathway, and therefore the more severe the drug cravings and the greater the probability of relapse. In other words, re-entering the drug-taking environment with a strong sense of agency should mimic the effects of having had a more extended drug-taking history.

## 5 Simultaneous manipulations of agency and affect

A popular model assumes that during the recovery process, individuals with substance-use disorder transition through five stages: *precontemplation*, during which there is little or no interest in change; *contemplation*, when change is first considered; *preparation* for change, when decisions are made and planning begins; *action*, when specific steps are taken to implement a recovery plan; and *maintenance*, when the new abstinence behaviors are consolidated and preserved (DiClemente et al., 2004; Prochaska and DiClemente, 1982). ADDS predicts that increases in the sense of agency will have different effects during these different stages of recovery.

Increases in agency facilitate many everyday actions, including for example, motivation, executive function, and procedural or skill learning. Ashby et al. (2024) identified the specialized phenomena of perceptual priming and standard eyeblink conditioning as rare examples that seem unaffected by changes in agency. Even so, importantly, they did not identify any behaviors that are adversely affected by increases in agency. So under non-addicted conditions, a high level of agency confers many benefits and few, if any costs.

ADDS predicts that behaviors associated with addiction sharply contrast with this more natural state of affairs. A high sense of agency will benefit abstinence during the maintenance stage of recovery, primarily because it will increase motivation and improve inhibitory self-control. But ADDS also predicts that increases in agency will accelerate the path to addiction during the pre-contemplation stage and make relapse more likely before maintenance is consolidated. First, as we saw in an earlier section, a higher sense of agency should accelerate the transition to addiction by increasing the motivation to find and take the drug and by increasing the synaptic plasticity that mediates drug-related learning and the development of the antireward response. And second, the previous section described how a higher sense of agency should exacerbate cravings, especially cravings that result when the drug-taking environment is revisited. Therefore, as discussed in the next section, ADDS recommends that after a commitment to recovery has been made, but before maintenance is consolidated, manipulations of agency during treatment for addiction should be nuanced—in recovery-reinforcing environments, recovery should be optimized if treatment increases sense of agency, whereas in addiction-reinforcing environments, cravings and relapse should be minimized if treatment decreases sense of agency. Unfortunately though, reducing agency in these conditions comes at a considerable cost—namely, motivation and inhibitory self-control will also be minimized. ADDS predicts that this cost is inevitable. Even so, some of these deleterious effects might be reduced by elevating the client's affect.

Many years ago, Ashby et al. (1999) proposed that events that induce mild positive affect elevate cortical DA levels for a period of 20–30 min, and that this DA increase facilitates all types of executive function, including inhibitory self-control. The idea is that events that elevate mood frequently include an unexpected reward. For example, a mild elevation in mood might occur after receiving an unexpected gift, or unexpectedly running into an old friend. DA neurons in the VTA fire phasically to unexpected rewards (Schultz et al., 1997), thereby raising DA levels in all VTA target regions, including the prefrontal cortex. After VTA DA neurons fire a phasic burst, DA levels in frontal cortex remain elevated for a period of 20–30 min. This is because cortex has only negligible concentrations of DA active transporter, which quickly clears DA from synapses (in contrast to basal ganglia, where concentrations are high; e.g., Varrone and Halldin, 2014). As a result, in cortex, free DA either eventually diffuses away from the synapse or is slowly degraded by the enzyme catechol-O-methyltransferase (COMT). Either way, following a phasic burst of DA neuron firing, DA levels remain elevated in cortex for much longer than, for example, in the basal ganglia. Furthermore, the evidence is good that these elevated cortical DA levels facilitate executive function (e.g., Ashby et al., 2015; Ott and Nieder, 2019), and as this theory predicts, so does mild positive affect (Ashby et al., 1999).

The theory that mild positive affect elevates cortical DA levels was proposed specifically to account for human behavior. Even so, there are now many results that extend that theory to nonhuman animals and specifically to the neuroanatomical models described in Figures 1 and 2. A complete review is beyond the scope of this article, but two illustrative supporting results are as follows. First, DA neurons in the VTA of mice increase their activity during social play (Solié et al., 2022), as predicted by the theory if one accepts that social play improves affect. Second, tickling adolescent rats causes DA increases in the nucleus accumbens and also elicits ultrasonic vocalizations (i.e., 50,000 Hz; Hori et al., 2013) that are thought to be indicators of positive affect (Knutson et al., 2002). These results support the assumption that events that induce mild positive affect also increase the firing of DA neurons in the VTA.

**Figure 3:**
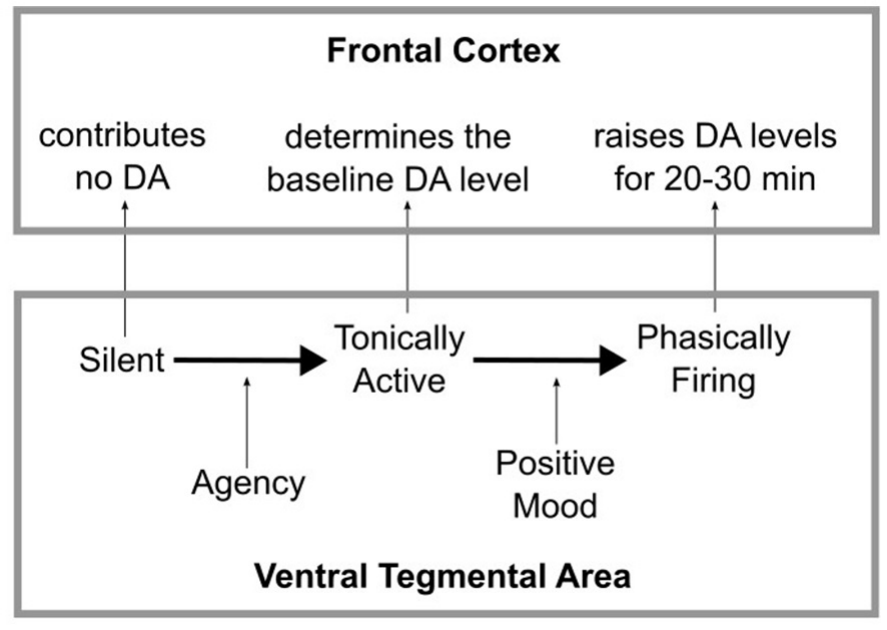
The three states of DA neurons: silent, tonically active, and phasically firing.

We now have three theoretical predictions, which all have considerable empirical support, that when combined might provide enhanced therapeutic interventions for treatment of substance-use disorders. First, ADDS predicts that increases in agency will increase the population of tonically active DA neurons in the VTA, but not increase the firing rate of those DA neurons that are tonically active. Second, the Ashby et al. (1999) theory predicts that events that induce mild positive affect will increase the firing rate of VTA DA neurons. Third, the evidence suggests that only tonically active DA neurons can transition to phasic firing (Grace et al., 2007; Lodge and Grace, 2006). In other words, VTA DA neurons silenced by tonic inhibition from the ventral pallidum should be incapable of responding to events that induce mild positive affect. The model that results when these three proposals are combined is illustrated in Figure 3. DA neurons in the VTA will typically be in one of three different states. First, because of inhibition from the ventral pallidum (see Figure 1), they could be completely silent, in which case they contribute no DA to any target areas, including frontal cortex. Second, they could be firing tonically (with typical firing rates of 0.2–10 Hz; Liu et al., 2021), thereby contributing to a steady influx of DA to frontal cortex that defines the baseline level of DA. Third, they could be firing phasically in response to an excitatory input (with a firing rate greater than 10 Hz). Even a short phasic burst will raise DA levels in frontal cortex above baseline for a period of 20–30 min (e.g., Feenstra and Botterblom, 1996). Importantly though, as we have seen, only those DA neurons that are already tonically active can transition to phasic firing (Grace et al., 2007; Lodge and Grace, 2006). Silent DA neurons must transition to tonic firing before they can respond to excitatory input with a phasic burst. As a result, ADDS predicts that increases in agency and elevations in affect have unique effects on the DA system.

Increases in agency should increase the number of tonically active DA neurons, but not cause phasic firing, because it should reduce tonic inhibition of the VTA DA neurons but not provide any new excitatory input. In contrast, events that induce positive affect should increase phasic firing in tonically active DA neurons, but have no effect on the total number that are tonically active. So agency facilitates the transition from silent to tonically active, whereas positive affect facilitates the transition from tonically active to phasic firing. As a result, following any therapeutic intervention that reduces agency, a subsequent manipulation that induces positive affect should maximize the benefits of the available tonically active DA neurons, without exacerbating the deleterious effects of place conditioning.

## 6 Treatment and recovery

Once a person with substance-use disorder commits to recovery, ADDS predicts that manipulations in the client's sense of agency should have potent therapeutic benefits. This section explores the ADDS predictions for treatment and recovery, and makes some recommendations about how traditional therapies might be improved.

### 6.1 Tests of ADDS predictions

ADDS predicts that treatment of substance-use disorders should be facilitated by appropriate changes to the client's sense of agency. The most therapeutic approach will depend on the client's stage of recovery. Although we have seen that changes in the sense of agency affect many aspects of human behavior, in the case of substance-use disorders, the most relevant effects are probably on motivation, executive function, and cue- and place-induced craving. A prominent cognitive theory proposes that executive function includes three core components: working memory, inhibitory self-control, and mental set shifting (Miyake et al., 2000). These are likely all important for recovery, but of the three, inhibitory self-control seems especially critical because such inhibition is needed to prevent relapse.

First, consider the pre-contemplation stage of recovery, during which the individual is more motivated to find and take the drug than to abstain and recover. During this stage, an increase in agency at any time should increase drug use because it will increase motivation as well as craving and compulsion. Thus, interventions that reduce sense of agency are recommended.

Second, during the maintenance stage, the primary motivation is now to abstain from drug taking and at this stage of recovery, the individual has been drug free long enough that any cravings that could be exacerbated by a high sense of agency should be absent, or at least highly reduced compared to earlier stages of recovery (e.g., Galloway et al., 2010). As a result, the most efficacious treatments will likely increase agency at all times in order to increase motivation and improve inhibitory self-control.

Third, consider the intervening stages during which the primary motivation is to recover but maintenance has not yet fully consolidated. During these stages, a high sense of agency will increase motivation to remain drug free, but also exacerbate the place- and cue-induced cravings that characterize these stages. As a result, the best approach might be to elevate sense of agency at some times and weaken it at others. In particular, sense of agency should be elevated during times when the individual is in a recovery-reinforcing environment that lacks drug-related cues—such as during inpatient rehabilitation, support-group meetings, or individual outpatient treatment. An elevated sense of agency during these periods should have many benefits, including for example, to increase motivation and improve inhibitory self-control, without the concomitant cost of amplified cravings. In contrast, ADDS also predicts that sense of agency should be reduced when an individual recovering from substance-use disorder is in an addiction-reinforcing environment—that is, one rich in cues that might induce cravings—such as a bar that serves alcohol, a drug dealer's house, or any setting where the individual has previously taken drugs. As we have seen, because of place conditioning, re-entering the drug-taking environment during a period of withdrawal or recovery will increase activation on the vSub → NAcc pathway, which will exacerbate drug craving and relapse. For these reasons, cravings and relapse should be minimized if the drug-taking environment is revisited when agency is low.

We know of no direct tests of these predictions. Even so, there is abundant supporting anecdotal evidence. First, many popular and successful therapeutic approaches include practices that are likely to increase sense of agency while the client is in a recovery-reinforcing environment; even if this is not a stated goal of the practice. In fact, Gorlin and Békés (2021) argued that a central (although typically unstated) goal of many different psychotherapeutic approaches is to increase the client's sense of agency, and the different approaches do this in a variety of ways, but one method used by almost all approaches is to improve the client's awareness of themselves and their environment. A number of recent studies have confirmed that sense of agency increases with self-knowledge and self-awareness (e.g., Braun et al., 2018; Gupta et al., 2025; Matsumiya, 2021). Different psychotherapeutic approaches use different methods to improve awareness. For example, (1) cognitive behavioral therapy uses psychoeducation to improve the client's awareness of the connections between their thoughts and emotions and their behavior (McHugh et al., 2010); (2) motivational interviewing encourages clients to believe that they can change and helps clients see the discrepancy between their current behaviors and their desired goals (Miller and Rollnick, 2002; Smedslund et al., 2011); (3) insight-oriented psychotherapy focuses on building a client's insight into how their past experiences affect their current behavior (Jennissen et al., 2018; Lysaker et al., 2015); and (4) acceptance and commitment therapy uses mindfulness-based practices, which have been shown to increase agency (Carlson, 2013; Lush et al., 2016), and to improve the client's understanding and awareness of their current emotions and thoughts (Bowen et al., 2009; Harris, 2019; Korecki et al., 2020). ADDS could therefore be viewed as providing a neuroscientific rationale for these diverse therapeutic interventions.

A high level of agency is generally considered to be a positive trait and a desired outcome of psychotherapy (Bandura, 1977). As a result, there is little or no literature investigating whether reductions in agency could ever be psychotherapeutic. For this reason, we know of no studies that have tested the ADDS prediction that sense of agency should be reduced when a person recovering from substance-use disorder visits an addiction-reinforcing environment. Given this, it is especially impressive that a variety of addiction therapies have simultaneously converged on a set of practices that are likely to do exactly this. For example, a common conversation in case conceptualization among treatment providers for individuals diagnosed with substance-use disorder focuses on the client's appropriate level of care. The consensus is that level of care should increase with the severity of the addiction—ranging from hospitalization for the most severe cases to semi-regular outpatient treatment for the least severe (Hoffman et al., 1993). The balance in this decision is about autonomy and support. Increasing support by increasing a client's level of care will also decrease the client's autonomy because of the increased restrictions that are necessary to provide that support. Autonomy is defined as “the ability to make your own decisions without being controlled by anyone else” (Cambridge Dictionary, n.d.). Therefore, the reduced autonomy that comes with increasing level of care should also reduce sense of agency.

Other common practices explicitly reduce sense of agency, without necessarily using that language. For example, Step 3 of the 12 steps to recovery recommended by Alcoholics Anonymous is “Made a decision to turn our will and our lives over to the care of God as we understood Him” (Alcoholics Anonymous, n.d.). As another example, it is common to ensure that a sober companion accompanies the recovering individual when they first revisit a triggering environment. Agency is likely to be reduced in such cases because studies show that taking an action that is directed by others (e.g., following orders) results in lower agency than if the same action is freely taken without any external direction (Caspar et al., 2016).

All current practices that serve to reduce agency in addiction-reinforcing environments most likely evolved as methods to control behavior—specifically, to decrease the likelihood of drug taking—rather than to decrease agency *per se*. Note that any such practice is complicated by the existing evidence that reductions in agency decrease inhibitory self-control. As a result, these practices tend to rely on some external constraints to prevent drug taking—such as hospitalization or ensuring that a sober person accompanies the recovering individual when they revisit a drug-taking environment. ADDS provides a neuroscientific justification for these practices. But it also predicts that reduced agency will have other benefits—specifically, a lessening of the drug cravings and compulsions that develop because of place conditioning.

### 6.2 Therapeutic recommendations

As we have seen, ADDS predicts that there may be therapeutic benefit to manipulating sense of agency in the appropriate direction at certain times during treatment of substance-use disorders. A reduced sense of agency could be beneficial at any time for clients in the pre-contemplation stage, and when clients in the contemplation, preparation, or action stages are visiting addiction-reinforcing environments. In contrast, an increased sense of agency could be beneficial at any time during maintenance, and when clients in the contemplation, preparation, or action stages are visiting recovery-reinforcing environments. Therefore, treatment will likely be facilitated if the treatment provider has available a repertoire of methods for both increasing *and* decreasing agency. ADDS makes no predictions about what those methods might look like, or about how they might be developed. This section makes a few suggestions, but these should be considered speculative, since they are well outside the scope of the current theory. As a result, more research on this problem is clearly needed.

Many methods of psychotherapy are rooted in psychological theories that predict that increases in agency are beneficial to psychological health. This list includes, for example, self-efficacy theory (Bandura, 1977), hope theory (Snyder, 1989), goal-focused positive psychotherapy (Conoley and Scheel, 2017), and self-determination theory (Deci and Ryan, 2000). As a result, many methods have been proposed for how a psychotherapist might increase a client's sense of agency. These methods might be especially important in the treatment of addiction because evidence suggests that drug use is associated with a lower overall sense of agency (Render and Jansen, 2019). As examples of the methods that have been proposed, Bandura (2012) identified four approaches that might be used to boost agency, or more specifically, what he called self-efficacy: (1) by increasing the client's mastery with respect to their ability to revisit addiction-reinforcing environments without relapse (i.e., each success should increase agency), (2) by encouraging clients to make their own choices, (3) via social modeling—that is, by observing success in others, and (4) via social (e.g., verbal) persuasion. These methods play a key role in the relapse prevention model of treatment proposed by Marlatt and Gordon (2005). Similarly, within goal-focused positive psychotherapy, a popular method to increase agency is via capitalization—that is, by celebrating client strengths (e.g., a healthy desire, behavior, or thought; Gable et al., 2018).

Not surprisingly, there are very few, if any, established methods with the stated goal of reducing a client's sense of agency. What sorts of treatment protocols might serve to lower agency? A few have already been mentioned, including for example, (1) ensuring that a sober companion accompanies the client during early revisits to drug-taking environments; (2) ensuring that the client attends regular meetings of a support group; and (3) moving the client to a sober-living environment. Some of these methods are practical only during initial periods of recovery. For example, ensuring that a sober companion accompanies the individual during revisits to drug-taking environments is not a practical long-term solution. What approaches might be used when accompaniment is no longer possible? We make two tentative suggestions—both of which could be used in conjunction with each other. However, neither method has received empirical testing, so clearly, more research is needed on this problem.

One method that might be used to reduce agency in individuals in later stages of substance-use disorder is to instruct them to use vivid visual imagery to imagine that the therapist or a sober companion is accompanying them when they revisit a drug-taking environment. Imagery and perception activate similar neural pathways (e.g., Dijkstra et al., 2019), so the neurological effects of imagining a companion should be similar to the effects produced by a real companion. In addition, imagining a helpful companion is a natural act. For example, psychotherapeutic clients often construct internalized representations of their psychotherapist that include auditory, visual, and kinesthetic components (Knox, 2003; Knox et al., 1999). Furthermore, evidence suggests that such internal representations are beneficial to the healing process (e.g., Knox, 2003). Therefore, the act of imagining a companion during a revisit to a drug-taking environment might have similar benefits to bringing along a real companion.

A second method to reduce agency under these conditions might be to instruct the client to write a script in advance of the visit that describes the behaviors they subsequently will follow during the visit. As in a script for a movie or stage play, the script should include a detailed description of behaviors that move the client through the environment during the visit. This method should reduce agency because agency is reduced when one's actions are directed rather than freely taken without any external guidance (Caspar et al., 2016).

Methods that reduce agency during visits to environments where drugs were previously taken should reduce cravings, but as previously described, there are likely costs. The most critical are probably reduced motivation to abstain from drug taking and reduced executive function—for example, reduced inhibitory self-control. As a result, any method of reducing these costs without also increasing agency is likely to have therapeutic benefit. One way to compensate might be to increase the client's affect via some unexpected intervention. For example, the therapist might send the client some text message just before the visit that temporarily improves the client's mood. As described earlier, this elevated mood should improve motivation and executive function for a period of 20–30 min, without exacerbating cravings.

## 7 Limitations

There are at least three significant limitations to consider before translating the predictions and suggestions made here to a clinical setting. First, all of the predictions derived in this article come from ADDS, which was developed only recently. Although the neuroscience underpinnings of this model (described in Figure 1) have received rigorous testing, the behavioral predictions of ADDS have not. As described here and by Ashby et al. (2024), there is considerable empirical support for many of these predictions, but even so, this support comes almost exclusively from studies that were not designed to test an ADDS prediction. As a result, much more empirical testing is needed to validate and/or improve the theory.

Second, many of the predictions derived here are based on research with nonhuman animals. Generalizing such results to humans requires caution because of differences in neuroanatomy—especially between rodents and humans, and because many of the predictions discussed in this article are about internal states that are unobservable in nonhuman animals, including for example, agency, craving, compulsion, and positive affect.

Third, even the human studies that were used to derive ADDS predictions mostly come from controlled laboratory experiments. As a result, caution must be used when attempting to interpret how such results might be extended to a real-world clinical setting.

## 8 Conclusions

Sense of agency critically affects many human behaviors and it is widely considered to play a key role in psychological health (e.g., Bandura, 1977). The ADDS theory proposes that increases in agency disinhibit the DA system and thereby increase the number of tonically active DA neurons in the ventral tegmental area. The theory makes many novel predictions about how sense of agency impacts all aspects of drug addiction. It also suggests a variety of novel addiction treatments, including for example, that the most therapeutic approach might be to elevate sense of agency at some times and weaken it at others. Furthermore, the theory predicts that reducing the agency of an individual in the contemplation, preparation, or action stages of recovery from substance-use disorder while they are revisiting a drug-taking environment will not only reduce drug-taking behaviors, but it will also reduce drug cravings, and therefore improve the psychological state of the individual.

## Data Availability

The original contributions presented in the study are included in the article/supplementary material, further inquiries can be directed to the corresponding author.

## Footnotes

1 (Inglis et al. 2021) made no assumptions about the psychological construct that was modulating vSub output. Specifically, they made no mention of agency, nor did they make any attempt to account for behavioral data. The hypothesis that agency is the key driver of this network was added by (Ashby et al. 2024).

2 By potentiating the glutamate response through NMDA receptors and depressing the glutamate response through non-NMDA receptors (Ashby and Casale, 2003).

3 Note that in the 17% of rats in which cocaine self-administration meets the criteria of a compulsion, drug seeking persists despite these negative hedonics.

4 There is also evidence for a role of the closely related ventral CA1 region of hippocampus in drug-related place conditioning (Zhou et al., 2019).

## References

[B1] Alcoholics Anonymous (n.d.). Step 3. In aa.org website. Available online at: https://www.aa.org/the-twelve-steps (Accessed February 10, 2025).

[B2] American Psychiatric Association (2022). Diagnostic and Statistical Manual of Mental Disorders, fifth edition, text revision. Washington, DC: American Psychiatric Association. doi: 10.1176/appi.books.9780890425787

[B3] Anthony J. C. Warner L. A. Kessler R. C. (1994). Comparative epidemiology of dependence on tobacco, alcohol, controlled substances, and inhalants: basic findings from the National Comorbidity Survey. Exp. Clin. Psychopharmacol. 2, 244–268. doi: 10.1037/1064-1297.2.3.244

[B4] Ashby F. G. Casale M. B. (2003). A model of dopamine modulated cortical activation. Neural Netw. 16, 973–984. doi: 10.1016/S0893-6080(03)00051-0 14692632

[B5] Ashby F. G. Isen A. M. Turken A. (1999). A neuropsychological theory of positive affect and its influence on cognition. Psychol. Rev. 106, 529–550. doi: 10.1037/0033-295X.106.3.529 10467897

[B6] Ashby F. G. Valentin V. V. von Meer S. S. (2015). Differential effects of dopamine-directed treatments on cognition. Neuropsychiatr. Dis. Treat. 11, 1859–1875. doi: 10.2147/NDT.S65875 26251602 PMC4524582

[B7] Ashby F. G. Zetzer H. A. Conoley C. W. Pickering A. (2024). Just do it: a neuropsychological theory of agency, cognition, mood, and dopamine. J. Exper. Psychol. 153, 1582–1604. doi: 10.1037/xge0001587 38884963

[B8] Badrinarayan A. Wescott S. A. Vander Weele C. M. Saunders B. T. Couturier B. E. Maren S. . (2012). Aversive stimuli differentially modulate real-time dopamine transmission dynamics within the nucleus accumbens core and shell. J. Neurosci. 32, 15779–15790. doi: 10.1523/JNEUROSCI.3557-12.2012 23136417 PMC3752139

[B9] Bandura A. (1977). Self-efficacy: toward a unifying theory of behavioral change. Psychol. Rev. 84, 191–215. doi: 10.1037/0033-295X.84.2.191 847061

[B10] Bandura A. (2006). Toward a psychology of human agency. Perspect. Psychol. Sci. 1, 164–180. doi: 10.1111/j.1745-6916.2006.00011.x 26151469

[B11] Bandura A. (2012). On the functional properties of perceived self-efficacy revisited. J. Manage. 38, 9–44. doi: 10.1177/0149206311410606

[B12] Bossert J. M. Adhikary S. St. Laurent R. Marchant N. J. Wang H.-L. Morales M. . (2016). Role of projections from ventral subiculum to nucleus accumbens shell in context-induced reinstatement of heroin seeking in rats. Psychopharmacology 233, 1991–2004. doi: 10.1007/s00213-015-4060-5 26344108 PMC4781679

[B13] Bossert J. M. Stern A. L. (2014). Role of ventral subiculum in context-induced reinstatement of heroin seeking in rats. Addict. Biol. 19, 338–342. doi: 10.1111/adb.12015 23231571 PMC3622847

[B14] Bowen S. Chawla N. Collins S. E. Witkiewitz K. Hsu S. Grow J. . (2009). Mindfulness-based relapse prevention for substance use disorders: a pilot efficacy trial. Substance Abuse 30, 295–305. doi: 10.1080/08897070903250084 19904665 PMC3280682

[B15] Braun N. Debener S. Spychala N. Bongartz E. Sörös P. Müller H. H. . (2018). The senses of agency and ownership: a review. Front. Psychol. 9:535. doi: 10.3389/fpsyg.2018.00535 29713301 PMC5911504

[B16] Broderick P. Gardner E. Van Praag H. (1984). In vivo electrochemical and behavioral evidence for specific neural substrates modulated differentially by enkephalin in rat stimulant stereotypy and locomotion. Biol. Psychiatry 19, 45–54. 6538442

[B17] Caban Rivera C. Price R. Petrilli Fortuna R. Li C. Do C. Shinkle J. . (2025). The ventral hippocampus and nucleus accumbens as neural substrates for cocaine contextual memory reconsolidation. Transl. Psychiatry 16:2. doi: 10.1038/s41398-025-03734-4 41271626 PMC12783833

[B18] Cambridge Dictionary (n.d.). Autonomy. In dictionary.cambridge.org dictionary. Available online at: https://dictionary.cambridge.org/dictionary/english/autonomy (Accessed February 10, 2025).

[B19] Carlson E. N. (2013). Overcoming the barriers to self-knowledge: mindfulness as a path to seeing yourself as you really are. Perspect. Psychol. Sci. 8, 173–186. doi: 10.1177/1745691612462584 26172498

[B20] Caspar E. A. Christensen J. F. Cleeremans A. Haggard P. (2016). Coercion changes the sense of agency in the human brain. Curr. Biol. 26, 585–592. doi: 10.1016/j.cub.2015.12.067 26898470 PMC4791480

[B21] Chiken S. Nambu A. (2016). Mechanism of deep brain stimulation: inhibition, excitation, or disruption? Neuroscientist 22, 313–322. doi: 10.1177/1073858415581986 25888630 PMC4871171

[B22] Conoley C. W. Scheel M. J. (2017). Goal Focused Positive Psychotherapy: A Strengths-Based Approach. Oxford: Oxford University Press. doi: 10.1093/med-psych/9780190681722.001.0001

[B23] de Jong J. W. Afjei S. A. Dorocic I. P. Peck J. R. Liu C. Kim C. K. . (2019). A neural circuit mechanism for encoding aversive stimuli in the mesolimbic dopamine system. Neuron 101, 133–151. doi: 10.1016/j.neuron.2018.11.005 30503173 PMC6317997

[B24] De Ridder D. Manning P. Glue P. Cape G. Langguth B. Vanneste S. (2016). Anterior cingulate implant for alcohol dependence: case report. Neurosurgery 78, E883–E893. doi: 10.1227/NEU.0000000000001248 27077594

[B25] De Ridder D. Vanneste S. Kovacs S. Sunaert S. Dom G. (2011). Transient alcohol craving suppression by rtms of dorsal anterior cingulate: an fMRI and LORETA EEG study. Neurosci. Lett. 496, 5–10. doi: 10.1016/j.neulet.2011.03.074 21458537

[B26] Deci E. L. Ryan R. M. (2000). The “what” and “why” of goal pursuits: human needs and the self-determination of behavior. Psychol. Inq. 11, 227–268. doi: 10.1207/S15327965PLI1104_01

[B27] Deroche-Gamonet V. Belin D. Piazza P. V. (2004). Evidence for addiction-like behavior in the rat. Science 305, 1014–1017. doi: 10.1126/science.1099020 15310906

[B28] Di Chiara G. Imperato A. (1988). Drugs abused by humans preferentially increase synaptic dopamine concentrations in the mesolimbic system of freely moving rats. Proc. Nat. Acad. Sci. 85, 5274–5278. doi: 10.1073/pnas.85.14.5274 2899326 PMC281732

[B29] DiClemente C. C. Schlundt D. Gemmell L. (2004). Readiness and stages of change in addiction treatment. Am. J. Addict. 13, 103–119. doi: 10.1080/10550490490435777 15204662

[B30] Dijkstra N. Bosch S. E. van Gerven M. A. (2019). Shared neural mechanisms of visual perception and imagery. Trends Cogn. Sci. 23, 423–434. doi: 10.1016/j.tics.2019.02.004 30876729

[B31] Drevets W. C. Savitz J. Trimble M. (2008). The subgenual anterior cingulate cortex in mood disorders. CNS Spectr. 13, 663–681. doi: 10.1017/S1092852900013754 18704022 PMC2729429

[B32] Feenstra M. G. Botterblom M. H. (1996). Rapid sampling of extracellular dopamine in the rat prefrontal cortex during food consumption, handling and exposure to novelty. Brain Res. 742, 17–24. doi: 10.1016/S0006-8993(96)00945-6 9117391

[B33] French S. J. Hailstone J. C. Totterdell S. (2003). Basolateral amygdala efferents to the ventral subiculum preferentially innervate pyramidal cell dendritic spines. Brain Res. 981, 160–167. doi: 10.1016/S0006-8993(03)03017-8 12885437

[B34] Gable S. L. Reis H. T. Impett E. A. Asher E. R. (2018). “What do you do when things go right? The intrapersonal and interpersonal benefits of sharing positive events,” in Relationships, well-being and behaviour, eds. Reis H. T. (London: Routledge). doi: 10.4324/9780203732496-6 15301629

[B35] Galloway G. P. Singleton E. G. Buscemi R. Baggott M. J. Dickerhoof R. M. Mendelson J. E. . (2010). An examination of drug craving over time in abstinent methamphetamine users. Am. J. Addict. 19, 510–514. doi: 10.1111/j.1521-0391.2010.00082.x 20958846

[B36] Gardner E. L. Lowinson J. H. (1993). Drug craving and positive/negative hedonic brain substrates activated by addicting drugs. Semin. Neurosci. 5, 359–368. doi: 10.1016/S1044-5765(05)80044-2

[B37] Gorlin E. I. Békés V. (2021). Agency via awareness: a unifying meta-process in psychotherapy. Front. Psychol. 12:698655. doi: 10.3389/fpsyg.2021.698655 34335416 PMC8316855

[B38] Grace A. A. (2010). Dopamine system dysregulation by the ventral subiculum as the common pathophysiological basis for schizophrenia psychosis, psychostimulant abuse, and stress. Neurotox. Res. 18, 367–376. doi: 10.1007/s12640-010-9154-6 20143199 PMC2932888

[B39] Grace A. A. Floresco S. B. Goto Y. Lodge D. J. (2007). Regulation of firing of dopaminergic neurons and control of goal-directed behaviors. Trends Neurosci. 30, 220–227. doi: 10.1016/j.tins.2007.03.003 17400299

[B40] Gupta K. V. Beuria J. Vijanapalli L. K. Sethi A. Behera L. (2025). Self-reflection, sense of agency, and underlying neural correlates: a pilot study. PLoS ONE 20:e0335276. doi: 10.1371/journal.pone.0335276 41452904 PMC12742732

[B41] Hamani C. Mayberg H. Stone S. Laxton A. Haber S. Lozano A. M. (2011). The subcallosal cingulate gyrus in the context of major depression. Biol. Psychiatry 69, 301–308. doi: 10.1016/j.biopsych.2010.09.034 21145043

[B42] Hand L. J. Paterson L. M. Lingford-Hughes A. R. (2024). Re-evaluating our focus in addiction: emotional dysregulation is a critical driver of relapse to drug use. Transl. Psychiatry 14:467. doi: 10.1038/s41398-024-03159-5 39521844 PMC11550421

[B43] Harris R. (2019). ACT Made Simple: An Easy-to-Read Primer on Acceptance and Commitment Therapy. Oakland, CA: New Harbinger Publications.

[B44] Hoffman N. G. Halikas J. A. Mee-Lee D. (1993). ASAM Patient Placement Criteria for the Treatment of Psychoactive Substance Use Disorders. Washington, DC: American Society of Addiction Medicine.

[B45] Hong S. Hikosaka O. (2014). Pedunculopontine tegmental nucleus neurons provide reward, sensorimotor, and alerting signals to midbrain dopamine neurons. Neuroscience 282, 139–155. doi: 10.1016/j.neuroscience.2014.07.002 25058502 PMC4302061

[B46] Hong S. Jhou T. C. Smith M. Saleem K. S. Hikosaka O. (2011). Negative reward signals from the lateral habenula to dopamine neurons are mediated by rostromedial tegmental nucleus in primates. J. Neurosci. 31, 11457–11471. doi: 10.1523/JNEUROSCI.1384-11.2011 21832176 PMC3315151

[B47] Hori M. Shimoju R. Tokunaga R. Ohkubo M. Miyabe S. Ohnishi J. . (2013). Tickling increases dopamine release in the nucleus accumbens and 50 kHz ultrasonic vocalizations in adolescent rats. Neuroreport 24, 241–245. doi: 10.1097/WNR.0b013e32835edbfa 23399995

[B48] Inglis J. B. Valentin V. V. Ashby F. G. (2021). Modulation of dopamine for adaptive learning: a neurocomputational model. Comput. Brain Behav. 4, 34–52. doi: 10.1007/s42113-020-00083-x 34151186 PMC8210637

[B49] Jay T. M. (2003). Dopamine: a potential substrate for synaptic plasticity and memory mechanisms. Prog. Neurobiol. 69, 375–390. doi: 10.1016/S0301-0082(03)00085-6 12880632

[B50] Jennissen S. Huber J. Ehrenthal J. C. Schauenburg H. Dinger U. (2018). Association between insight and outcome of psychotherapy: systematic review and meta-analysis. Am. J. Psychiatry 175, 961–969. doi: 10.1176/appi.ajp.2018.17080847 30068262

[B51] Karin O. Raz M. Alon U. (2021). An opponent process for alcohol addiction based on changes in endocrine gland mass. IScience 24:102127. doi: 10.1016/j.isci.2021.102127 33665551 PMC7903339

[B52] Knox S. (2003). I sensed you with me the other day: a review of the theoretical and empirical literature on clients' internal representations of therapists. College Educ. Fac. Res. Public. 99, 1–7. doi: 10.1037/e565752006-001

[B53] Knox S. Goldberg J. L. Woodhouse S. S. Hill C. E. (1999). Clients' internal representations of their therapists. J. Couns. Psychol. 46, 244–256. doi: 10.1037/0022-0167.46.2.244

[B54] Knutson B. Burgdorf J. Panksepp J. (2002). Ultrasonic vocalizations as indices of affective states in rats. Psychol. Bull. 128, 961–977. doi: 10.1037/0033-2909.128.6.961 12405139

[B55] Kobayashi Y. Okada K. (2007). Reward prediction error computation in the pedunculopontine tegmental nucleus neurons. Ann. N. Y. Acad. Sci. 1104, 310–323. doi: 10.1196/annals.1390.003 17344541

[B56] Koob G. F. Le Moal M. (2008a). Addiction and the brain antireward system. Annu. Rev. Psychol. 59, 29–53. doi: 10.1146/annurev.psych.59.103006.093548 18154498

[B57] Koob G. F. Le Moal M. (2008b). Neurobiological mechanisms for opponent motivational processes in addiction. Philos. Trans. R. Soc. B 363, 3113–3123. doi: 10.1098/rstb.2008.0094 PMC2607326 18653439

[B58] Korecki J. R. Schwebel F. J. Votaw V. R. Witkiewitz K. (2020). Mindfulness-based programs for substance use disorders: a systematic review of manualized treatments. Subst. Abuse Treat. Prev. Policy 15:51. doi: 10.1186/s13011-020-00293-3 32727559 PMC7392831

[B59] Li X. Hartwell K. J. Borckardt J. Prisciandaro J. J. Saladin M. E. Morgan P. S. . (2013). Volitional reduction of anterior cingulate cortex activity produces decreased cue craving in smoking cessation: a preliminary real-time fMRI study. Addict. Biol. 18, 739–748. doi: 10.1111/j.1369-1600.2012.00449.x 22458676 PMC3389595

[B60] Liu C. Goel P. Kaeser P. S. (2021). Spatial and temporal scales of dopamine transmission. Nat. Rev. Neurosci. 22, 345–358. doi: 10.1038/s41583-021-00455-7 33837376 PMC8220193

[B61] Lodge D. J. Grace A. A. (2006). The hippocampus modulates dopamine neuron responsivity by regulating the intensity of phasic neuron activation. Neuropsychopharmacology 31, 1356–1361. doi: 10.1038/sj.npp.1300963 16319915

[B62] Lodge D. J. Grace A. A. (2008). Amphetamine activation of hippocampal drive of mesolimbic dopamine neurons: a mechanism of behavioral sensitization. J. Neurosci. 28, 7876–7882. doi: 10.1523/JNEUROSCI.1582-08.2008 18667619 PMC2562638

[B63] Lüscher C. Janak P. H. (2021). Consolidating the circuit model for addiction. Annu. Rev. Neurosci. 44, 173–195. doi: 10.1146/annurev-neuro-092920-123905 33667115

[B64] Lush P. Parkinson J. Dienes Z. (2016). Illusory temporal binding in meditators. Mindfulness 7, 1416–1422. doi: 10.1007/s12671-016-0583-z 27909466 PMC5107189

[B65] Lysaker P. H. Kukla M. Belanger E. White D. A. Buck K. D. Luther L. . (2015). Individual psychotherapy and changes in self-experience in schizophrenia: a qualitative comparison of patients in metacognitively focused and supportive psychotherapy. Psychiatry 78, 305–316. doi: 10.1080/00332747.2015.1063916 26745684

[B66] Ma Y.-Y. Lee B. R. Wang X. Guo C. Liu L. Cui R. . (2014). Bidirectional modulation of incubation of cocaine craving by silent synapse-based remodeling of prefrontal cortex to accumbens projections. Neuron 83, 1453–1467. doi: 10.1016/j.neuron.2014.08.023 25199705 PMC4295617

[B67] Malagon-Vina H. Ciocchi S. Klausberger T. (2023). Firing patterns of ventral hippocampal neurons predict the exploration of anxiogenic locations. Elife 12:e83012. doi: 10.7554/eLife.83012 37039474 PMC10089657

[B68] Marchant N. J. Campbell E. J. Whitaker L. R. Harvey B. K. Kaganovsky K. Adhikary S. . (2016). Role of ventral subiculum in context-induced relapse to alcohol seeking after punishment-imposed abstinence. J. Neurosci. 36, 3281–3294. doi: 10.1523/JNEUROSCI.4299-15.2016 26985037 PMC4792939

[B69] Marlatt G. A. Gordon J. R. (2005). Relapse Prevention: Maintenance Strategies in the Treatment of Addictive Behaviors. New York: Guilford Press.

[B70] Matsumiya K. (2021). Awareness of voluntary action, rather than body ownership, improves motor control. Sci. Rep. 11:418. doi: 10.1038/s41598-020-79910-x 33432104 PMC7801649

[B71] Matsumoto M. Hikosaka O. (2007). Lateral habenula as a source of negative reward signals in dopamine neurons. Nature 447, 1111–1115. doi: 10.1038/nature05860 17522629

[B72] Matsumoto M. Hikosaka O. (2009). Representation of negative motivational value in the primate lateral habenula. Nat. Neurosci. 12, 77–84. doi: 10.1038/nn.2233 19043410 PMC2737828

[B73] McGlinchey E. M. James M. H. Mahler S. V. Pantazis C. Aston-Jones G. (2016). Prelimbic to accumbens core pathway is recruited in a dopamine-dependent manner to drive cued reinstatement of cocaine seeking. J. Neurosci. 36, 8700–8711. doi: 10.1523/JNEUROSCI.1291-15.2016 27535915 PMC4987439

[B74] McHugh R. K. Hearon B. A. Otto M. W. (2010). Cognitive-behavioral therapy for substance use disorders. Psychiatr. Clin. North Am. 33, 511–525. doi: 10.1016/j.psc.2010.04.012 20599130 PMC2897895

[B75] McKendrick G. Graziane N. M. (2020). Drug-induced conditioned place preference and its practical use in substance use disorder research. Front. Behav. Neurosci. 14:582147. doi: 10.3389/fnbeh.2020.582147 33132862 PMC7550834

[B76] Mele A. R. (2003). Motivation and Agency. Oxford: Oxford University Press. doi: 10.1093/019515617X.001.0001

[B77] Metcalfe J. Greene M. J. (2007). Metacognition of agency. J. Exper. Psychol. 136, 184–199. doi: 10.1037/0096-3445.136.2.184 17500645

[B78] Miller W. R. Rollnick S. (2002). Motivational Interviewing: Preparing People for Change. New York: Guilford Press.

[B79] Miyake A. Friedman N. P. Emerson M. J. Witzki A. H. Howerter A. Wager T. D. (2000). The unity and diversity of executive functions and their contributions to complex “frontal lobe” tasks: a latent variable analysis. Cogn. Psychol. 41, 49–100. doi: 10.1006/cogp.1999.0734 10945922

[B80] Moore J. W. (2016). What is the sense of agency and why does it matter? Front. Psychol. 7:1272. doi: 10.3389/fpsyg.2016.01272 27621713 PMC5002400

[B81] Moorman D. E. Aston-Jones G. (2023). Prelimbic and infralimbic medial prefrontal cortex neuron activity signals cocaine seeking variables across multiple timescales. Psychopharmacology 240, 575–594. doi: 10.1007/s00213-022-06287-2 36464693 PMC10406502

[B82] Moser M.-B. Rowland D. C. Moser E. I. (2015). Place cells, grid cells, and memory. Cold Spring Harb. Perspect. Biol. 7:a021808. doi: 10.1101/cshperspect.a021808 25646382 PMC4315928

[B83] Nazzaro J. Seeger T. Gardner E. (1981). Morphine differentially affects ventral tegmental and substantia nigra brain reward thresholds. Pharmacol. Biochem. Behav. 14, 325–331. doi: 10.1016/0091-3057(81)90398-1 7232458

[B84] Nett K. E. Zimbelman A. R. McGregor M. S. Vera V. A. Harris M. R. LaLumiere R. T. (2023). Infralimbic projections to the nucleus accumbens shell and amygdala regulate the encoding of cocaine extinction learning. J. Neurosci. 43, 1348–1359. doi: 10.1523/JNEUROSCI.2023-22.2022 36657972 PMC9987566

[B85] Niv Y. Daw N. D. Joel D. Dayan P. (2007). Tonic dopamine: opportunity costs and the control of response vigor. Psychopharmacology 191, 507–520. doi: 10.1007/s00213-006-0502-4 17031711

[B86] O'Brien C. P. Childress A. R. McLellan A. T. Ehrman R. (1992). Classical conditioning in drug-dependent humans. Ann. N. Y. Acad. Sci. 654, 400–415. doi: 10.1111/j.1749-6632.1992.tb25984.x 1632593

[B87] Okada K. Kobayashi Y. (2013). Reward prediction-related increases and decreases in tonic neuronal activity of the pedunculopontine tegmental nucleus. Front. Integr. Neurosci. 7:36. doi: 10.3389/fnint.2013.00036 23717270 PMC3653103

[B88] O'Mara S. (2006). Controlling hippocampal output: The central role of subiculum in hippocampal information processing. Behav. Brain Res. 174, 304–312. doi: 10.1016/j.bbr.2006.08.018 17034873

[B89] Ott T. Nieder A. (2019). Dopamine and cognitive control in prefrontal cortex. Trends Cogn. Sci. 23, 213–234. doi: 10.1016/j.tics.2018.12.006 30711326

[B90] Prochaska J. O. DiClemente C. C. (1982). Transtheoretical therapy: toward a more integrative model of change. Psychotherapy 19, 276–288. doi: 10.1037/h0088437

[B91] Randall P. A. McElligott Z. A. Besheer J. (2020). Role of mPFC and nucleus accumbens circuitry in modulation of a nicotine plus alcohol compound drug state. Addict. Biol. 25:e12782. doi: 10.1111/adb.12782 31173443 PMC6898730

[B92] Render A. Jansen P. (2019). Dopamine and sense of agency: determinants in personality and substance use. PLoS ONE 14:e0214069. doi: 10.1371/journal.pone.0214069 30889224 PMC6424396

[B93] Robbins T. W. Banca P. Belin D. (2024). From compulsivity to compulsion: the neural basis of compulsive disorders. Nat. Rev. Neurosci. 25, 313–333. doi: 10.1038/s41583-024-00807-z 38594324

[B94] Russell J. (2013). Agency: Its Role in Mental Development. New York: Psychology Press. doi: 10.4324/9780203775691

[B95] Salamone J. D. Cousins M. S. Bucher S. (1994). Anhedonia or anergia? Effects of haloperidol and nucleus accumbens dopamine depletion on instrumental response selection in a T-maze cost/benefit procedure. Behav. Brain Res. 65, 221–229. doi: 10.1016/0166-4328(94)90108-2 7718155

[B96] Schultz W. Dayan P. Montague P. R. (1997). A neural substrate of prediction and reward. Science 275, 1593–1599. doi: 10.1126/science.275.5306.1593 9054347

[B97] Shaham Y. Shalev U. Lu L. De Wit H. Stewart J. (2003). The reinstatement model of drug relapse: history, methodology and major findings. Psychopharmacology 168, 3–20. doi: 10.1007/s00213-002-1224-x 12402102

[B98] Smedslund G. Berg R. C. Hammerstrøm K. T. Steiro A. Leiknes K. A. Dahl H. M. . (2011). Motivational interviewing for substance abuse. Campbell System. Rev. 7, 1–126. doi: 10.4073/csr.2011.6 PMC8939890 21563163

[B99] Snyder C. R. (1989). Reality negotiation: from excuses to hope and beyond. J. Soc. Clin. Psychol. 8, 130–157. doi: 10.1521/jscp.1989.8.2.130

[B100] Sobstyl M. Kupryjaniuk A. Prokopienko M. Rylski M. (2022). Subcallosal cingulate cortex deep brain stimulation for treatment-resistant depression: a systematic review. Front. Neurol. 13:780481. doi: 10.3389/fneur.2022.780481 35432155 PMC9012165

[B101] Solié C. Girard B. Righetti B. Tapparel M. Bellone C. (2022). VTA dopamine neuron activity encodes social interaction and promotes reinforcement learning through social prediction error. Nat. Neurosci. 25, 86–97. doi: 10.1038/s41593-021-00972-9 34857949 PMC7612196

[B102] Solomon R. L. Corbit J. D. (1974). An opponent-process theory of motivation: I. Temporal dynamics of affect. Psychol. Rev. 81, 119–145. doi: 10.1037/h0036128 4817611

[B103] Taepavarapruk P. Butts K. A. Phillips A. G. (2015). Dopamine and glutamate interaction mediates reinstatement of drug-seeking behavior by stimulation of the ventral subiculum. Int. J. Neuropsychopharmacol. 18, 1–9. doi: 10.1093/ijnp/pyu008 25539503 PMC4368862

[B104] Taepavarapruk P. Phillips A. G. (2003). Neurochemical correlates of relapse to d-amphetamine self-administration by rats induced by stimulation of the ventral subiculum. Psychopharmacology 168, 99–108. doi: 10.1007/s00213-002-1337-2 12655460

[B105] Tian J. Uchida N. (2015). Habenula lesions reveal that multiple mechanisms underlie dopamine prediction errors. Neuron 87, 1304–1316. doi: 10.1016/j.neuron.2015.08.028 26365765 PMC4583356

[B106] Varrone A. Halldin C. (2014). “Human brain imaging of dopamine transporters,” in Imaging of the Human Brain in Health and Disease, eds. Seeman P., and Madras B. (Amsterdam: Elsevier), 203–240. doi: 10.1016/B978-0-12-418677-4.00009-9

[B107] Volkow N. D. Fowler J. S. Wang G.-J. Swanson J. M. Telang F. (2007). Dopamine in drug abuse and addiction: results of imaging studies and treatment implications. Arch. Neurol. 64, 1575–1579. doi: 10.1001/archneur.64.11.1575 17998440

[B108] Vorel S. R. Liu X. Hayes R. J. Spector J. A. Gardner E. L. (2001). Relapse to cocaine-seeking after hippocampal theta burst stimulation. Science 292, 1175–1178. doi: 10.1126/science.1058043 11349151

[B109] Zhao D. Zhang X. Bai J. (2025). Medial prefrontal cortex circuit dynamics involved in stage-specific addiction. Brain Struct. Funct. 230:185. doi: 10.1007/s00429-025-03055-8 41307570

[B110] Zhao Y. Sallie S. N. Cui H. Zeng N. Du J. Yuan T. . (2021). Anterior cingulate cortex in addiction: new insights for neuromodulation. Neuromodulation 24, 187–196. doi: 10.1111/ner.13291 33090660

[B111] Zhou Y. Zhu H. Liu Z. Chen X. Su X. Ma C. . (2019). A ventral CA1 to nucleus accumbens core engram circuit mediates conditioned place preference for cocaine. Nat. Neurosci. 22, 1986–1999. doi: 10.1038/s41593-019-0524-y 31719672

